# Band Structure Extraction at Hybrid Narrow‐Gap Semiconductor–Metal Interfaces

**DOI:** 10.1002/advs.202003087

**Published:** 2020-12-31

**Authors:** Sergej Schuwalow, Niels B. M. Schröter, Jan Gukelberger, Candice Thomas, Vladimir Strocov, John Gamble, Alla Chikina, Marco Caputo, Jonas Krieger, Geoffrey C. Gardner, Matthias Troyer, Gabriel Aeppli, Michael J. Manfra, Peter Krogstrup

**Affiliations:** ^1^ Center for Quantum Devices Niels Bohr Institute University of Copenhagen and Microsoft Quantum Materials Lab Copenhagen Lyngby Denmark; ^2^ Paul Scherrer Institut Swiss Light Source PSI Villigen CH‐5232 Switzerland; ^3^ Microsoft Quantum One Microsoft Way Redmond WA 98052 USA; ^4^ Microsoft Station Q Purdue Birck Nanotechnology Center Purdue University West Lafayette IN 47907 USA; ^5^ Department of Physics and Astronomy Purdue University West Lafayette IN 47907 USA; ^6^ Physics Department ETH Zurich CH‐8093 Switzerland; ^7^ Institut de Physique EPFL Lausanne CH‐1015 Switzerland; ^8^ School of Electrical and Computer Engineering and School of Materials Engineering Purdue University West Lafayette IN 47907 USA

**Keywords:** angle‐resolved photoelectron spectroscopy, Hybrid interfaces, Majorana zero modes, quantum devices, semiconductors, topological superconductors

## Abstract

The design of epitaxial semiconductor–superconductor and semiconductor–metal quantum devices requires a detailed understanding of the interfacial electronic band structure. However, the band alignment of buried interfaces is difficult to predict theoretically and to measure experimentally. This work presents a procedure that allows to reliably determine critical parameters for engineering quantum devices; band offset, band bending profile, and number of occupied quantum well subbands of interfacial accumulation layers at semiconductor‐metal interfaces. Soft X‐ray angle‐resolved photoemission is used to directly measure the quantum well states as well as valence bands and core levels for the InAs(100)/Al interface, an important platform for Majorana‐zero‐mode based topological qubits, and demonstrate that the fabrication process strongly influences the band offset, which in turn controls the topological phase diagrams. Since the method is transferable to other narrow gap semiconductors, it can be used more generally for engineering semiconductor–metal and semiconductor–superconductor interfaces in gate‐tunable superconducting devices.

## Graphical abstract

Detailed understanding of band alignments and band bending profiles in metal–semiconductor heterostructures is critical for the development of quantum computing devices. This work describes a procedure for extraction of the band alignment in buried metal–semiconductor interfaces using angular resolved photoemission spectroscopy.

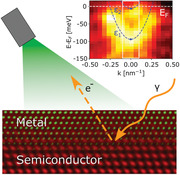

Semiconductor‐metal (SM) interfaces play a central role in modern electronic devices. Since the 1960s, efforts were made to obtain reliable predictions for their key electronic parameter, the band alignment, from charge neutrality points of metal‐induced gap states (MIGS),^[^ ^1^, ^2^ ^]^ defect levels,^[^ ^3^ ^]^ or interface reactions.^[^ ^4^ ^]^ Databases with material parameters specifying bulk properties of semiconductors and metals have existed in literature for decades. However, databases for interfaces are missing, partly because of the preparation‐dependent interface chemistry and partly because of limited characterization options. Here, we describe a new method to reliably determine key electronic parameters of narrow gap SM interfaces.

In this work, we use angle‐resolved photoelectron spectroscopy (ARPES), photon energy dependent core‐level spectroscopy, and self‐consistent electronic structure calculations to determine the band alignment of InAs(100)/Al epitaxial interfaces, which have recently gained interest for their potential in topological quantum computing.^[^ ^5^, ^6^, ^7^, ^8^, ^9^ ^]^ We demonstrate, given a measurement of the band offset at the InAs(100)/vacuum interface, how core‐level measurements can be used to analyze the band alignment and offset of buried SM interfaces based on the InAs substrate. Since the band offset is one of the most important parameters for the simulation of topological phase diagrams of Majorana‐zero‐modes in semiconductor nanowires,^[^ ^10^, ^11^ ^]^ this method will become a crucial tool for the targeted design of topological quantum bit devices. It may also be used to determine the quality of the interface by comparing the measured Fermi‐level pinning position with the energies of impurities and defects calculated from ab‐initio methods. In contrast to previous attempts to determine the band offset from core‐level or valence‐band spectroscopy,^[^ ^12^, ^13^, ^14^ ^]^ we show that our procedure correctly determines the number and energy of quantum well subbands at the interface, which demonstrates a significant advancement in the accuracy of the band offset extraction. We further discuss how this method is transferable to other important narrow gap SM interfaces, such as those based on InSb. Note that we perform the spectroscopic measurements at a temperature of T≈15K, above the superconducting transition temperature of aluminium. However, because the band offset is identical for the superconducting and normal state, this means that our results are transferable to devices operated at lower temperatures.

There are two generic scenarios for band bending at interfaces: In the simplest case, the Fermi‐level is pinned in the semiconductor band gap where low temperatures and in the absence of doping lead to a negligible band bending due to lack of free carriers. If, however, the Fermi‐level at the interface is pinned outside the gap, band bending is formed due to accumulation of carriers (either holes or electrons depending on the sign of the band offset).

**Figure** **1** exemplifies this for the InSb(110) and InAs(100) interfaces, respectively, as characterized by soft X‐ray angle‐resolved photoemission spectroscopy (SX‐ARPES). The Fermi‐level of the InSb(110) surface is pinned in the gap (Figure 1a). The valence band structure consists of the heavy‐hole (HH), light‐hole (LH), and split‐off bands (SO), with the valence band maximum (VBM) visible at ≈0.2 eV below the Fermi‐level (for details see Supporting Information: Methodology). In the absence of an accumulation layer and without a significant doping of the semiconductor substrate, we thus expect the band edge to have a flat depth profile on the length scale relevant for nanoscale device physics. This is consistent with previous reports for cleaved InSb(110) and InAs(110) surfaces that reported flat bands due to the absence of dangling bond surface states in the (indirect) band gap.^[^ ^15^, ^16^ ^]^

**Figure 1:**
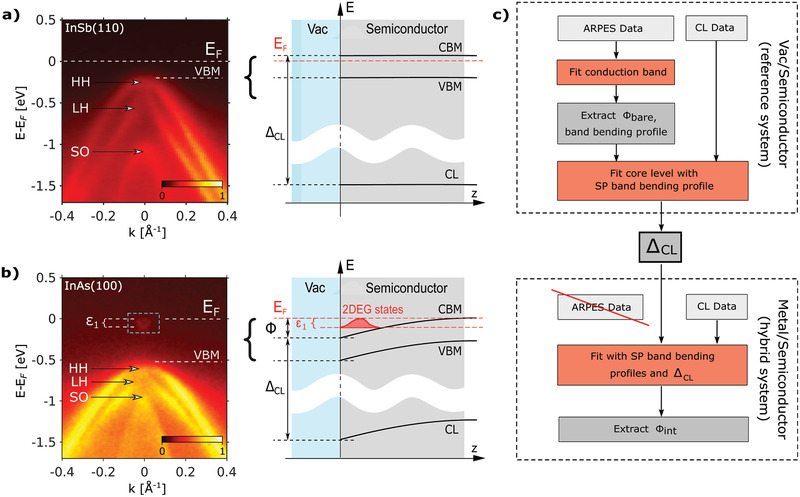
a) ARPES band structure (hν = 707 eV) and band alignment diagram for a system with surface Fermi level pinning within the gap, here exemplified for InSb(110). b) Same as (a) but for InAs(100), a system with the Fermi‐level pinned above the conduction band minimum (CBM) and strong band bending (hν = 405 eV). $\varepsilon_{1}$ denotes the visible offset of the first QW subband relative to the Fermi‐level $E_{F}$, with the usual abbreviations for valence band maximum (VBM), heavy‐hole (HH), light‐hole (LH), and split‐off (SO) bands. Emergent 2DEG states are highlighted. c) Flow diagram of the band offset determination procedure. The three fitting steps are highlighted in red. We emphasize that only core level data is needed for the investigation of the buried hybrid interfaces.

In contrast, the pristine InAs(100) surface (Figure 1b) is known to exhibit a downward band bending toward the surface.^[^ ^17^, ^18^ ^]^ The Fermi‐level is pinned in the conduction band and the resulting quantum well contains a 2D electron gas (2DEG) confined to the surface. The quantum well states are visible just below the Fermi‐level with subband energies $\varepsilon_{i}$ at the Γ point (at k=0) where *i* is the subband number. Φ denotes the conduction band offset. Because of the additional energy increase due to confinement the bottom of the lowest quantum well, subband $\varepsilon_{1}$ is not equal to the band offset Φ.

The usual approach to extract band offsets from photoemission spectra is to either measure the distance of the VBM to the Fermi‐level (which also gives the conduction band offset by adding the band gap energy), or to measure the binding energy of a core level (CL) if its energetic separation to the VBM is known.^[^ ^13^, ^14^, ^19^, ^20^, ^21^ ^]^ The VBM is determined using the intersection between the linear fit of the background in the band gap^[^ ^22^ ^]^ and the extrapolation of the valence band leading edge. However, comparison between the band offset determined by this approach and a direct measurement of the quantum well state subband occupation and energies have generally shown poor agreement and suggests systematic errors in this method.^[^ ^12^, ^13^ ^]^ Such errors can have various origins: Even for flat bands, accurate determination of the VBM is not straightforward, especially in the case of narrow band gap semiconductors, because the background in the gap can be nonlinear due to band broadening. In systems with band bending, the confining potential at the interface affects all photoemission spectra, both valence band and core levels, through asymmetric broadening and shifting of peak positions, especially in materials with narrow and deep quantum wells.^[^ ^12^, ^23^ ^]^ Deconvolution methods for these band‐bending modulated photoemission spectra were developed in the past^[^ ^24^, ^25^, ^26^, ^27^, ^28^, ^29^ ^]^ using analytical potential shapes or interface models. However, the typically large number of fitting parameters in such models often make this deconvolution procedure an ill‐posed mathematical problem. Additionally, band bending can be challenging to disentangle from other sources of line broadening, such as lifetime effects, instrumental resolution, and chemical shifts.^[^ ^30^ ^]^ Despite these challenges, core‐level spectroscopy of buried interfaces remains attractive due to the large photoemission cross‐section of core‐levels, which remain the only accessible spectroscopic features in the case of thick overlayers.

Here, we outline a new approach that avoids many of the systematic errors that we have mentioned above. It consists of two parts (see Figure 1c): 1) determination of an accurate value of the characteristic bulk energy difference between a CL and the CBM of the semiconductor $\Delta_{\mathrm{CL}}$, and 2) CL measurements of the semiconductor at the buried interface. The procedure involves three fitting^[^ ^31^ ^]^ steps which are described in detail in Supporting Information B and D.

We assume that all energy levels are uniformly affected by the potential profile, and the energy difference $\Delta_{\mathrm{CL}}$ is a property of the bulk material. Note that accurate values for $\Delta_{\mathrm{CL}}$ are typically not available in literature. We determine $\Delta_{\mathrm{CL}}$ by measuring conduction band spectra and CL spectra from the bare semiconductor, along with self‐consistent simulation of the corresponding band bending profiles. We use the Schrödinger–Poisson (S‐P) approach^[^ ^32^ ^]^ in this work; however, more involved approaches such as the k·p formalism (or many‐body methods) can also be used. It is well known from the literature that the S‐P approach is well suited to describe the relationship between band bending and quantum‐well subbands both in classical semiconductor devices, such as field‐effect transistors,^[^ ^33^ ^]^ as well as topological materials, such as topological insulators.^[^ ^34^ ^]^ Additionally, the S‐P approach is less computationally expensive than calculations based on density functional theory, which would require large supercells to accommodate band bending over large length scales. Moreover, such ab initio methods would also require knowledge about the details of interface reconstructions and interface chemistry which may be difficult to obtain experimentally. We note that additional emerging states associated with the interface (e.g., trivial or topological) will affect the interface density of states (DOS). Consequently, it will change the Fermi level pinning of the conduction band and cause a shift in the core‐level positions due to the change of the electrostatic potential. Thus, once $\Delta_{\mathrm{CL}}$ is known, only CL spectra are necessary for the determination of interface parameters. Here, we apply the method to planar InAs(100)/Al samples, where $\Delta_{\mathrm{CL}}$ is extracted from a InAs(100)/vacuum interface. The result of the fitting of the ARPES data in the vicinitiy of the Γ point (see Supporting Information A) is shown in **Figure** **2**. The two dashed blue lines in Figure 2a represent the quantum well subband dispersions, obtained from a fit of the data highlighted with a white rectangle. The corresponding energy distribution curve (EDC) for k=0 is shown in Figure 2b. Finite instrument resolution is explicitly included in the fitting, and the proximity to the Fermi level is responsible for a shift in the perceived subband peak energy $\varepsilon_{2}$, as shown in Figure 2c. (More details about the fitting procedure are given in Supporting Information B.) From the fitting of the subbands via a Schrödinger–Poisson calculation, we obtain a band offset of $\Phi_{InAs(100)}=-0.20\pm 0.01\mathrm{eV}$, with the subband energies ε ${}_{1}=-0.097\pm 0.003\mathrm{eV}$ and ε ${}_{2}=-0.006\pm 0.003\mathrm{eV}$, respectively. Note that here we decided to only fit the data close to the Γ point at k${}_{\left|\right|}=0$. This approach does not require to make any assumptions about the functional form of the band dispersion (which would require to introduce additional fitting parameters for the in‐plane effective mass and non‐parabolicity parameters), but is sufficient to extract the subband energies of the quantum well states, which are the only quantities needed to determine the band offset.

**Figure 2:**
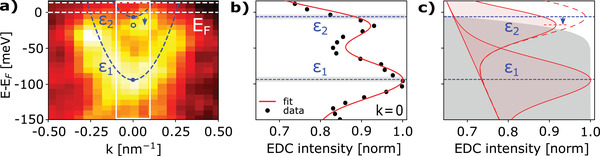
a) Zoom‐in of the QW states highlighted in Figure 1b (hν = 405 eV). The blue dashed lines show the dispersions of the lowest two QW states in a Schrödinger‐Poisson model fit to the data, which yields the band offset ϕ=−0.20±0.01. The fit area is shown as a white rectangle. b) Energy distribution curve (EDC) for the experimental spectrum shown in a) at k=0. Black dots represent the data. The red solid line shows the SP fit, with the EDC profile described by two Gaussian functions with linear background. Dashed blue lines indicate the energy levels $\varepsilon_{1,2}$ obtained from the SP fit and grey areas show their 1σ confidence regions. c) Shift in the subband energy ε2 due to finite instrument resolution and signal attenuation by proximity to the Fermi level. Attenuation effect on a unit signal shown in the background as a gray area.

The corresponding core level data are in **Figure** **3**. They are recorded at the same sample position as the angle resolved spectra using a photon energy range of 350–1050 eV (Figure 3a). The ratio of bulk to surface contribution varies with photon energy because of the energy‐dependent escape depth of the emitted electrons. This allows us to separate the surface and bulk contributions of the core levels, and to capture the trend of the band bending. Any well‐defined core‐level can be chosen for this procedure depending on the material—here, we use the In4d core level. Figure 3a shows the core level data set for the InAs(100)/vacuum interface. The shape of the In4d level in InAs has been subject to discussions in the literature in the past.^[^ ^17^, ^35^ ^]^ It is known that the spectral line consists of two distinct components, each exhibiting a two‐peak shape caused by the spin–orbit interaction; a main component originating from the bulk and a smaller contribution stemming from the surface layer of the material, which is shifted in energy due to the different local environment and bond formation. The bulk‐like component contains information about the band bending profile. The core level profiles in the entire energy range are simultaneously fitted using the potential profile obtained from the Schrödinger–Poisson simulation of the quantum well subbands, assuming a rigid energy shift (Supporting Information D). The plausible basis for this assumption is that the built‐in field is simultaneously affecting all states of the spectrum. From the fitting, we obtain $\Delta_{CL}=-17.22\pm 0.015\mathrm{eV}$ for the InAs In4d core level.

**Figure 3:**
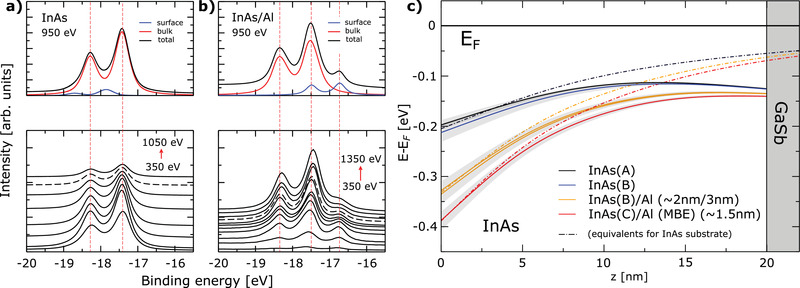
a) In4d core level data for photon energies between 350 and 1050 eV for the pristine InAs surface (Sample A). The top panel shows the decomposition of the In4d level into the surface and bulk components. The bottom panel shows the raw data, shifted vertically for convenience. The dataset shown in the top panel is highlighted with a dashed line. b) Same, but for InAs with ≈2 nm of Al deposited inside the ARPES chamber (Sample B), shown for photon energies between 350 and 1350 eV. c) Band bending potentials for the InAs(100)/Vac and InAs(100)/Al interfaces. Same‐sample results are color‐coded, with 1σ confidence intervals shown in the background. Full lines correspond to the 20nm InAs slab used in the experimental setup. Dashed lines show equivalent calculations assuming semi‐infinite InAs slab.

After extracting $\Delta_{CL}$ from the InAs(100)/vacuum interface, we can use this information to characterize the InAs(100)/Al heterostructure. Figure 3b shows the change in the In4d line shape upon Al deposition. The most immediate effect is the emergence of a distinct three‐peak structure, with the additional component now located at lower binding energies than the bulk feature. Analysis of the relative intensity modulation between the surface and bulk components (Supporting Information C) reveals that this low‐energy component can be attributed to traces of metallic In located on top of the deposited Al layer. This behavior is consistent with the relative weakness of In‐Al bonds, and the system minimizing energy by removing In from the surface in favor of As‐Al bond formation. InAl, to our knowledge, is not known to form any stochiometric compound, and the strongly different sizes of the atoms involved seem to result in a migration of excess In to the upper layers of the deposited Al thin film, similar to what has been previously observed for InSb/Al.^[^ ^36^, ^37^ ^]^ To investigate the influence of the fabrication process on the band alignment in InAs(100)/Al, we compare three different samples, a pristine InAs surface (Sample A) analyzed in Figure 2 that was prepared by decapping a protective amorphous overlayer, a separate sample where Al layers of varying thickness were deposited onto the freshly decapped InAs substrate directly inside the ARPES preparation chamber (Sample B, clean surface, and with ≈2/≈3 nm Al), and an MBE‐grown InAs/Al layer (Sample C, ≈1.5 nm Al). Since we know $\Delta_{\mathrm{CL},\mathrm{In}4d}$ for InAs and use physically self‐consistent profiles obtained by the SP approach, the fitting of the InAs(100)/Al core levels becomes a well‐defined mathematical problem with a single band parameter, the interface band offset $\Phi_{int}$.

Both the reference InAs fit and the results of the core level fitting procedure for the subsequent samples are shown in Figure 3c, along with their confidence regions. The majority of the error stems from the range of band offsets which produce a reasonable fit of the two confined states during the initial CB fitting of the reference sample. The pristine InAs surface demonstrates a downward band‐bending of approx. 0.2 eV, in line with the band bending strength suggested (but not observed in the CL data) by King et al.^[^ ^13^ ^]^ For the buried InAs/Al interface, the model clearly demonstrates a further increase of the band offset by approx. 0.1–0.2 eV. Note that the in situ MBE‐deposited Al film (C), which is known^[^ ^38^ ^]^ to produce very clean epitaxial interfaces, shows a comparatively stronger effect of Al deposition than the film deposited inside the ARPES chamber (B), which could be a consequence of higher substrate and interface quality. We do not detect a difference in the measured band bending profile between the 2 and 3 nm Al deposition in the ARPES chamber outside of our error margins. For a comparison, the dashed lines show the calculated band bending profiles for a semi‐infinite InAs slab instead of a 20 nm thick substrate used in the experiment. Note that the band offset of InAs(100)/Al is a local property of the interface and thus unaffected by the presence or absence of the GaSb substrate. The results are summarized in **Table** **1**.

To validate the aforementioned procedure, we perform a direct SX‐ARPES measurement of the MBE‐grown InAs(100)/Al sample (Sample C) on which the Al overlayer is thin enough to still allow access to the quantum well states (**Figure** **4**a). To compensate for the attenuation by the Al layer the measurement is performed at a higher photon energy hv=1045 eV, which results in an increased electron mean free path at the cost of reduced energy resolution. In Figure 4b, we zoom in on the conduction band QW states. The overlaid blue dashed lines are the QW states obtained independently by the core level fitting and self‐consistent SP approach. A comparison between these and a direct fit of the EDC, for the *k* = 0 bin, is displayed in Figure 4c. The core level fitting procedure predicts the energy levels of the first and second QW state as ε ${}_{1,CL}=-0.17\pm 0.01\mathrm{eV}$ and ε ${}_{2,CL}=-0.065\pm 0.01\mathrm{eV}$. These results are in good agreement with values obtained from a direct fitting ARPES data (ε ${}_{1,direct}=-0.166\pm 0.02\mathrm{eV}$ and ε ${}_{2_{,}direct}=-0.063\pm 0.02\mathrm{eV}$), even though the resolution of the quantum well states is low. The obtained band offsets of $\Phi_{int,CL}=-0.39\pm 0.02\mathrm{eV}$, and $\Phi_{int,direct}=-0.38\pm 0.04\mathrm{eV}$, respectively, confirm that the accuracy of the method is comparable with the accuracy obtained by direct fitting of the CB states (when accessible). We emphasize that the method described here only requires a sequence of core level measurements of the buried interface system. Given the high intensity of core‐level photoemission, we expect that an accurate determination of band offsets in buried interfaces under up to 6–8 nm of metallic overlayers should be possible when using soft X‐ray photons. There are two main reasons for the increased accuracy of the procedure presented here compared to previously employed methods that we have discussed above: first, the choice of a good reference system for the determination of $\Delta_{\mathrm{CL}}$ and the use of self‐consistent SP potentials in conjunction with $\Delta_{\mathrm{CL}}$ for the core level fitting. We suggest the conduction band states of an electron accumulation layer as the reference system because of their clear signature suitable for fitting and their well‐defined relation to the shape of the confining potential. In this case, it is convenient that an accumulation layer occurs naturally in the InAs(100) surface. If the bare surface does not have an accumulation layer, like in the case of InSb, it may be induced intentionally by surface doping as shown in refs. [39, 40, 41] for InAs and in ref. [42] in the case of InSb. Second, the use of self‐consistent SP potentials throughout the fitting procedure guarantees a physically sound relationship between band offset Φ and the band bending profile that enters the core level model (Supporting Information D). Because of this, the core line shape and the core level binding energy are no longer independent fitting parameters up to a fixed energy difference which is a material property of the bulk system ($\Delta_{\mathrm{CL}}$). This presents a very strong fitting constraint. If $\Delta_{\mathrm{CL}}$ is known, the offset Φ can be determined accurately, and vice versa.

**Table 1:** Overview of subband energies $\varepsilon_{1,2}$ and band offset parameters Φ for the investigated heterostructures, along with their 1σ errors

Material	Sample	$\varepsilon_{1}$/$\varepsilon_{2}$(err.) [eV]	Φ [eV]
InAs	A	−0.097/‐0.006(±0.003)	−0.20±0.01
InAs	B	−0.099/‐0.011(±0.007)	−0.21±0.02
InAs/Al (≈2 nm)	B	−0.147/‐0.049(±0.009)	−0.34±0.02
InAs/Al (≈3 nm)	B	−0.144/‐0.047(±0.009)	−0.33±0.02
InAs/Al (MBE) (≈1.5 nm)	C	−0.170/‐0.065(±0.01)	−0.39±0.02

**Figure 4:**
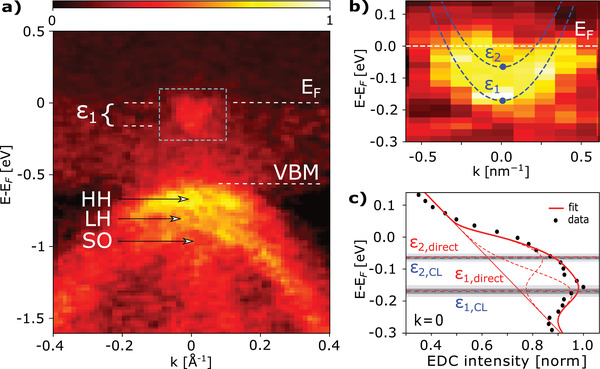
Verification by comparison with direct measurement. a) Background‐subtracted SX‐ARPES spectrum of the buried interfacial electronic structure of MBE grown InAs/Al(≈1.5nm) heterostructure (Figure 3c, Sample C) at hv = 1045 eV (compare with Figure 1b). b) A zoom in on the QW states of the InAs/Al system, overlaid with states calculated independently with input from *only* the core level fitting procedure. c) EDC for the InAs/Al experimental spectrum at *k* = 0. The red full/dashed lines shows a direct SP fit and the energy levels obtained therefrom (compare with Figure 2b); blue dashed lines show the energy levels obtained from the core level fitting procedure. Light and dark grey areas show their 1σ confidence regions, respectively.

In summary, the method described in this work can serve as a general approach to extract reliable values for: a) the characteristic bulk energy separation between conduction band and core level $\Delta_{\mathrm{CL}}$, and b) the band offsets Φ, the key parameter of SM interfaces in general, and a critical parameter for engineering hybrid quantum devices. While the direct measurements of the interface QWS subbands was used in this work as a verification of the core level fitting result (Figure 4), this step is not required. The core lines typically provide orders of magnitude higher photoemission signal as compared to conduction or valence band photoemission, which drastically reduces the acquisition times and/or lowers requirements as to overlayer thickness and surface quality. It further removes the need for angular resolution, thus increasing accessibility. The method can be transferred to all narrow gap semiconductors where an accumulation layer can be induced, for example, by alkali metal dosing on InSb/vacuum surfaces. The concept of this approach can also be extended to semiconductor heterostructures and other buried interfaces, however, noting that modifications to the analysis protocol are likely needed. We believe that the combined advantages of this approach can strongly contribute to rapid development of novel material combinations for targeted heterostructure interface design.

## Conflict of Interest

The authors declare no conflict of interest.

## Supporting information

Supporting Information
