# Supplementary material for: Band Structure Extraction at Hybrid Narrow‐Gap Semiconductor–Metal Interfaces
Source: Adv Sci (Weinh). 2020 Dec 31;8(4):2003087. doi: 10.1002/advs.202003087 (PMC7887586; doi:10.1002/advs.202003087)
Supplement: Supplementary file 1 — Supporting Information [file ADVS-8-2003087-s001.pdf]

© 2020 Wiley-VCH GmbH

ADVANCED SCIENCE
Open Access

# Supporting Information

for *Adv. Sci.*, DOI: 10.1002/advs.202003087

Band Structure Extraction at Hybrid Narrow-Gap Semiconductor-Metal Interfaces-Supplementary

*Sergej Schuwalow* Niels B. M. Schr  oter Jan Gukelberger Candice Thomas Vladimir Strocov John Gamble Alla Chikina Marco Caputo Jonas Krieger Geo_rey C. Gardner Matthias Troyer Gabriel Aeppli Michael J. Manfra Peter Krogstrup**

# Band Structure Extraction at Hybrid Narrow-Gap Semiconductor-Metal Interfaces - Supplementary

*Sergej Schuwalow* Niels B. M. Schröter Jan Gukelberger Candice Thomas Vladimir Strocov John Gamble Alla Chikina Marco Caputo Jonas Krieger Geoffrey C. Gardner Matthias Troyer Gabriel Aeppli Michael J. Manfra Peter Krogstrup**

*S.S. and N.B.M.S. contributed equally to this work*

## Methodology

The planar InAs-based materials were grown by molecular beam epitaxy on GaSb (100) substrates in a Veeco Gen 930 using ultra-high purity techniques and methods as described in [1, 2]. The structures are composed of a GaSb buffer grown at 500°C followed by a 20 nm-thick InAs layer grown at 480°C, as measured by absorption of blackbody radiation. The transition between these two materials has been made using a shutter sequence developed in [3]. The growth of InAs was performed with an As-to-In beam equivalent pressure ratio slightly larger than 1 to prevent the formation of void defects associated with As etching of the GaSb layer. Under these conditions, flat InAs surface morphology has been obtained with a roughness of the order of a monolayer. To facilitate the observation of the quantum well states, the first d=15 nm of the InAs layers were doped with Si atoms at a density $n_{Si,3D} = 2.2 \times 10^{18} cm^{-3}$. After the growth, the samples were covered with an amorphous arsenic cap layer deposited at 0°C to protect the InAs surface during transport in nitrogen atmosphere from the MBE-system in Purdue to the SX-ARPES system at the ADRESS beamline of the Swiss Light Source. The InAs samples were decapped by annealing at $\sim 350°$ C in the ultra-high vacuum system at ADRESS.
During the ARPES in-situ deposition steps described in the second part of the paper Al layers with different thicknesses were deposited at a temperature of T$\sim$15 K on top of the pristine InAs(100) by employing a shadow mask technique. The layer thickness was checked post deposition as a part of the fit described in Supplementary C.
The InSb(110) surface shown in Fig.1a of the main text was prepared by cleaving inside the ADRESS preparation chamber. We used an undoped n-type epi-ready InSb(110) wafer with a nominal carrier concentration of $5 \times 10^{13} - 3 \times 10^{14} cm^{-3}$.

## Supplementary A: Photon energy dependence of the photoemission signal.

It is well known from the literature (see e.g. Ref. [4]) that the intensity maxima for III-V direct band gap semiconductors should appear at a photon energy that corresponds to probing the Gamma point in the 3D Brillouin zone. To find the $\Gamma$ point for InAs(100), we varied the photon energy until we observed a strong intensity from the quantum well subbands, which is centered around $h\nu$=405 eV, as illustrated in Figure S1a. Note that around that energy, there is some contribution from core levels at the Fermi-level due to higher harmonics of the monochromator. We fine-tuned the photon energy to 405 eV to remove the core-level contribution from the energy window of interest. Since we were not able to detect any quantum well state emission for InSb(110), we identified the Gamma point from the valence band maximum, which is located at around $h\nu$=707 eV (see Figure S1b). This photon energy also corresponds to a high symmetry plane in the iso-energy surface shown in Figure S1c.

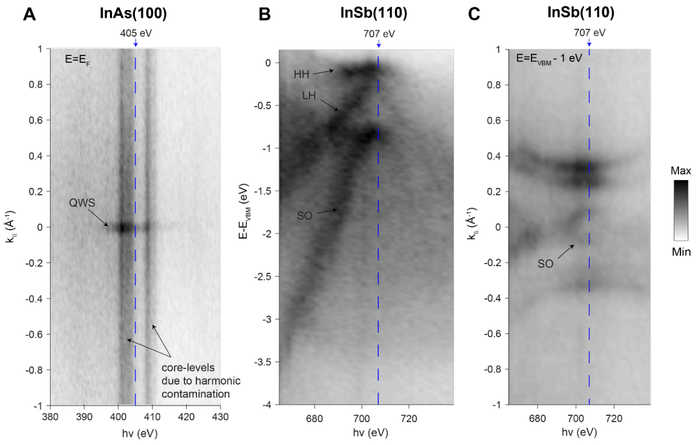


Figure S1: a) Photon energy dependence of the quantum well subbands (QWS) in InAs integrated 100 meV around the Fermi-level and measured around normal emission with linear-vertical polarization. b) Photon-energy dependent band dispersion measured in InSb(110) in normal-emission with linear-vertical polarization. Heavy-hole (HH), light-hole (LH), and split-off (SO) bands are indicated by black arrows. c) Photon energy dependence of the valence band emission integrated around a 100 meV energy window 1 eV below the valence band maximum. Split-off band forms a circle that is centred at a high-symmetry plane.

## Supplementary B: Schrödinger-Poisson fit of the conduction band spectrum

Electrostatic potential profile and subband energies are obtained from a self-consistent Schrödinger-Poisson (S-P) solver [5]. Herein, the Poisson equation $\epsilon\Delta\phi = -\rho$ for the electrostatic potential $\phi$ due to the electron charge density $\rho = \sum_n |\psi_n|^2$ is coupled with the Schrödinger equation $E_n\psi_n = \left[-\frac{\hbar^2}{2m_*^{\mathrm{InAs}}}\nabla^2 + (V - q\phi)\right]\psi_n$ for the quantum mechanical eigenvalue problem, wherein $E_n$ are the electron state energies, $\psi_n$ their corresponding wavefunctions, q is the electron charge, and V accommodates potential contributions beyond the electrostatic term $-q\phi$, which may include heterojunction potential steps and electronic exchange and correlation.

The conduction, heavy-hole and light-hole bands are included in the model. We include the InAs layer and a 100 nm thick slice of the GaSb substrate in the calculation, with space discretization along the growth direction $\Delta z \leq 0.2$ nm. The band offset $\Phi$ enters the Poisson equation as a Dirichlet boundary condition at the semiconductor surface, whereas a Neumann condition is imposed at the bottom of the system. Band structure parameters, including the conduction band effective mass $m_*^{\mathrm{InAs}} = 0.026$ and the InAs-GaSb valence band offset of 0.56 eV, are taken from [6]. Given a S-P solution, a predicted ARPES intensity profile is calculated by convolving the native signal $I_{\mathrm{int}}(E,k) = \left[\sum_{i=1,2} a_i \exp(-\frac{[E-\epsilon_i(k)]^2}{\sigma_{\mathrm{int}}^2})\right]\theta(-E) + a_{\mathrm{bg}}E + b_{\mathrm{bg}}$ with the known instrument resolution [1]. $\theta(-E)$ is the Fermi-Dirac step. Since the in-plane subband dispersions $\epsilon_{1,2}(k) = \epsilon_{1,2} + \frac{\hbar^2 k^2}{2m_*^{\mathrm{InAs}}}$ depend on the band offset $\Phi$ via the S-P solution, we obtain $\Phi$ along with all auxiliary parameters (subband intensities $a_{1,2}$, native linewidth $\sigma_{\mathrm{int}}$, and linear background $a_{\mathrm{bg}}$, $b_{\mathrm{bg}}$) from a single least-squares fit of the predicted intensities to the measured intensity profile. There are a total of 6 fitting parameters: the slope $a_{bg}$ and offset $b_{bg}$ of the linear background, the intrinsic width of the Gaussians $\sigma$ and their amplitudes $A_1$, $A_2$, and the band offset $\Phi$. The latter determines the two subband energies $\epsilon_1$,$\epsilon_2$ via the solution of a S-P calculation. Whilst the number of fitting parameters may appear large, we are still confident that our fitting produces the correct band offset. The reason is that the S-P solution strongly constrains the number of and separation between the Gaussian peaks (Figure S2c), which in turn puts constraints on their amplitude and linewidths. Thus, the fitting function and procedure is motivated by the known physics of the two-dimensional electron gas that can be described well with S-P calculations, which strongly limits the permissible parameter space and ensures physically reasonable solutions.

[1] We neglect higher subbands crossing the band offset only for band offsets $\Phi \gtrsim 0.6$ eV as well as Lorentzian broadening.

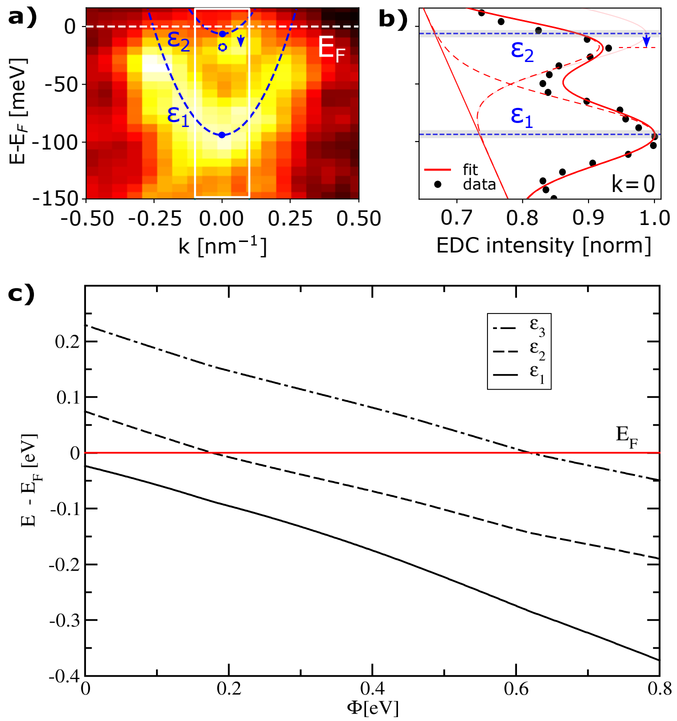


Figure S2: a),b) Effect of the proximity to the Fermi level on the conduction band fit shown in Figure 2 of the main text. The perceived maximum of the $\epsilon_2$ subband is adjusted downwards due to the finite resolution of the experimental setup. The underlying Gaussian function before accounting for finite resolution is shown as a light red line in b). c) Relationship between subband energies and band offset $\Phi$, calculated via S-P model of the InAs/GaSb slab.

Note that the energies of the perceived subband maxima close to the Fermi level can be affected by the finite instrument resolution (see Figure S2a, b), which is accounted for in the model. The perceived maximum of the second quantum well subband is slightly shifted from the energy $\epsilon_2$ to higher binding energies due to the effect of the multiplication with the Fermi-function and instrumental broadening. The underlying Gaussian function of the $\epsilon_2$ subband before accounting for finite resolution is shown as a light red line in Figure S2b.
To reiterate, the function of the conduction band fitting procedure is to obtain the band offset $\Phi$ (along with other auxiliary parameters) from the conduction band spectrum. This is used in the "reference system" step of the band offset determination procedure loop shown in Figure.1c of the main manuscript.

## Supplementary C: Origin of the shifted In4d core line component after Al deposition

Deposition of Al on InAs(100) gives rise to an increasingly distinct 3-peak structure of the In4d core level, as shown in Figure S3a for $h\nu$ =750 eV and 1/2/3nm Al, respectively. The origin of the shifted core line can be deduced by analyzing the signal for different energies and Al layer thickness. The main core level feature (red) is understood to originate from InAs proper, and thus its total intensity for a given photon energy $h\nu$ is expected to be well described by $\int_{surface}^{\infty} I_0 \exp\left(-z / \lambda[h\nu]\right) dz$, with $I_0$ some unit intensity and $\lambda$ the inelastic mean free path of electrons in InAs. To avoid discussion of superimposed intensity variations due to changes of the photon flux with different monochromator settings and the possible dependence of photoemission cross-section matrix elements on energy we renormalize the data to reproduce this behavior in the bulk component and compare the phenomenological behavior of the core-level sub feature with model predictions.
Figs. S3 b), c) show two different models for the intensities of the individual components of the In4d level as a function of photon energy $h\nu$. The symbols correspond to core line components shown in panel a). No reasonable agreement between experimental data and the model can be found under the assumption that the shifted In component originates from the InAs surface (Figure S3b). On the contrary, the

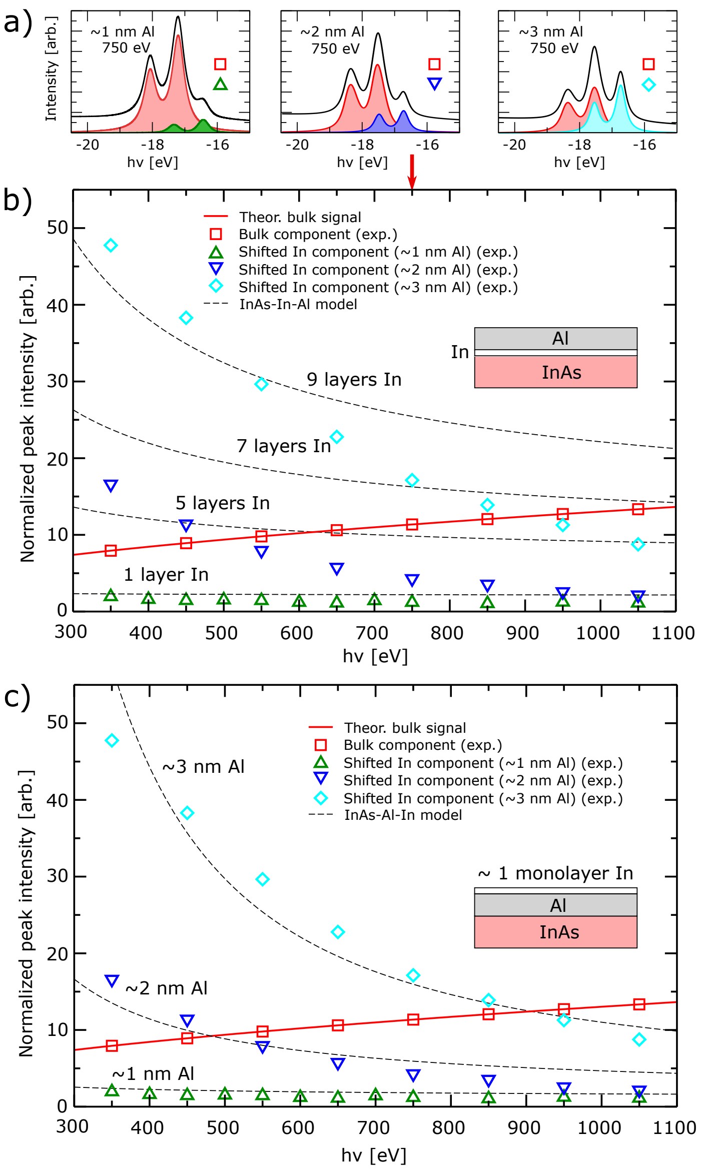


Figure S3: a) Phenomenological change in the In4d core level structure for Al deposition thickness of 1, 2 and 3 nm, here for h$\nu$ = 750 eV. Decomposition in bulk-like and shifted components is shown. b) Normalized intensity of the shifted In4d component vs. theoretical model under the assumption that the signal originates from indium at the InAs/Al interface (InAs-In-Al model). Dashed lines show theoretical intensities from different amounts of In at the interface. No reasonable agreement between model and experiment can be reached, even for unrealistic amounts of In. b) Same, but under the assumption that the shifted In core line originates from the top of the Al layer. Good agreement can be reached between theoretical intensities and experimental observation for a total amount of $\sim$ 1 monolayer of In migrating to the top of the Al layer during deposition.

data is readily explained by a total of ∼1 monolayer of In being removed from InAs and migrating to the top of the Al layer during Al deposition.

### Supplementary D: Core level fitting procedure for extraction of band bending profiles

To determine the band bending profiles shown in Figure 3c we simultaneously fit the core level shape of a set of spectra (∼ 10) in the 350-1350 eV range. The surface component for each photon energy $h\nu$ is represented by

$$
\begin{aligned}
S = \int_0^{d_{surf}} & I_0(h\nu) \times \exp(-z/\lambda) \times \\
& \times \Big\{ V_1(\alpha, \gamma, E - E_s) \\
& + \beta * V_2(\alpha, \gamma, E - E_s + SO) \Big\} \, dz
\end{aligned}
$$

whereas the the bulk component is represented by

$$
\begin{aligned}
B = \int_{d_{surf}}^{\infty} & I_0(h\nu) \times \exp(-z/\lambda) \times \\
& \times \Big\{ V_1(\alpha, \gamma, E + \Delta_{CL} + \phi(z)) \\
& + \beta * V_2(\alpha, \gamma, E + \Delta_{CL} + \phi(z) + SO) \Big\} \, dz
\end{aligned}
$$

and can be further attenuated by the presence of Al (compare Supplementary C). Here, V is a Voigt profile with $\alpha$ and $\gamma$ being the FWHM of the Gaussian and Lorentzian components, respectively, SO is the spin-orbit splitting and $E_s$ is the energy of the In4d surface component. $\phi(z)$ is the band bending potential, predicted by the SP model for a given band offset $\Phi$. The inelastic mean free path $\lambda$ is taken from the publications [7, 8, 9] where we use the fits to optical data, if available, and values given by the TPP-2M formula otherwise. The prefactor $I_0$ is dependent on energy and absorbs variations in flux, detector sensitivity and photoemission cross-section.

In total, for the In4d core level of the pristine semiconductor system there are 5 global fitting parameters for the entirety of the data: band offset $\Phi$, spin-orbit splitting SO, surface core level shift $E_s$, Lorentzian broadening of the core lines $\gamma$ and the branching ratio $\beta$. In addition, each data set has 3 local fitting parameters: the prefactor $I_0$ and the offset and slope of the background $a_{bg}$, $a_{bg}$. We obtain the Gaussian broadening $\alpha$ for each set from an independent resolution calculation. For the InAs/Al hybrid interface the Al layer thickness is included as an additional global fitting parameter.

The description of ∼ 10 data sets spanning the energy range between $350 - 1350$ eV using only 5(6) global parameters produces a robust core level model which allows us to cleanly separate surface and bulk contributions and extract the underlying value of the band offset $\Phi$ with a high degree of accuracy. To reiterate, the function of the core level fitting procedure is to obtain the value of $\Delta_{CL}$ if the band offset $\Phi$ (and thus the band bending profile $\phi(z)$) is known, *or* to obtain the band offset $\Phi$ if the value of $\Delta_{CL}$ is known. This is used in the "reference system" and "hybrid system" steps of the band offset determination procedure loop (Figure 1c of the main manuscript), respectively.

### Supplementary E: Universality of core level data

The In4d core level are used throughout this work because of their very well defined surface and bulk components which can be easily disentangled. For other systems other core lines can be used instead to the same effect. To highlight the universality of the approach in the Figure S4 we show the band offsets as recalculated using the As3d core levels instead. We obtain the characteristic energy difference $\Delta_{\mathrm{CL,\ As3d}} = -40.68 \pm 0.03$ eV for the As3d core level. The offsets lie within the error margins obtained in the original fit.

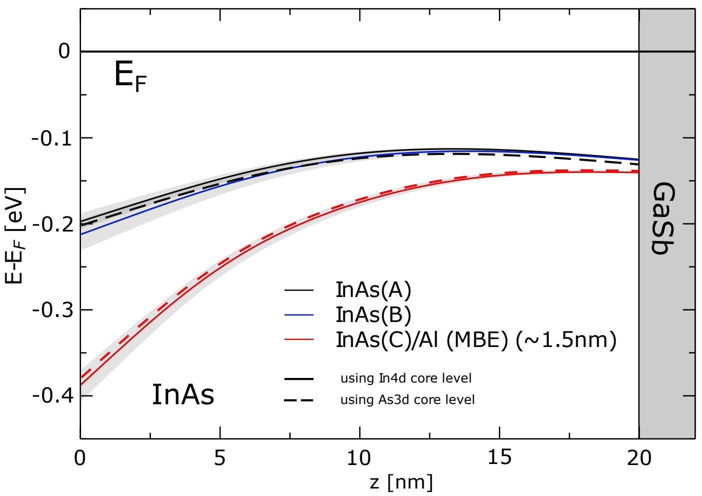


Figure S4: Comparison of the band offset extraction using In4d or As3d core levels.

# References

[1] G. C. Gardener, S. Falahi, J. D. Watson, M. J. Manfra, *Journal of Crystal Growth* **2016**, *441* 71.

[2] C. Thomas, A. T. Hatke, A. Tuaz, T. Wu, T. Wang, R. E. Diaz, G. C. Gardner, M. A. Capano, M. J. Manfra, *Phys. Rev. Mat.* **2018**, *2* 104602.

[3] G. Tuttle, H. Kroemer, J. H. English, *J. Appl. Phys.* **1990**, *67* 3032.

[4] L. O. Olsson, C. B. M. Andersson, M. C. Haakansson, J. Kanski, L. Ilver, U. O. Karlsson, *Phys. Rev. Lett.* **1996**, *76* 19.

[5] A. Trellakis, A. T. Galick, A. Pacelli, U. Ravaioli, *Journal of Applied Physics* **1997**, *81*, 12 7880.

[6] I. Vurgaftman, J. R. Meyer, L. R. Ram-Mohan, *Journal of Applied Physics* **2001**, *89*, 11 5815.

[7] S. Tanuma, C. J. Powell, D. R. Penn, *Surface and Interface Analysis* **1991**, *17* 927.

[8] S. Tanuma, C. J. Powell, D. R. Penn, *Surface and Interface Analysis* **1991**, *17* 911.

[9] S. Tanuma, C. J. Powell, D. R. Penn, *Surface and Interface Analysis* **1993**, *21* 165.
